# Altered immune responses in HR-HPV infected women

**DOI:** 10.3389/fmed.2026.1986999

**Published:** 2026-10-15

**Authors:** Cecilia Martínez-Campos, Kirvis Torres-Poveda, Marcela Lizano, Susana del Toro-Arreola, Carla O. Contreras-Ochoa, Margarita Bahena-Román, Víctor Hugo Bermúdez-Morales, Oscar Peralta-Zaragoza, Alfredo Lagunas-Martínez, Abrahan Ramírez-González, Jorge Cime-Castillo, Aurelio Cruz-Valdez, Vicente Madrid-Marina

**Affiliations:** 1 Subdirección de Desarrollo de Aplicaciones Clínicas, Instituto Nacional de Medicina Genómica, Mexico City, México; 2 Centro de Investigación Sobre Enfermedades Infecciosas, Instituto Nacional de Salud Pública, Cuernavaca, Morelos, México; 3 Subdirección de Investigación Básica, Instituto Nacional de Cancerología, Mexico City, Mexico; 4 Departamento de Medicina Genómica y Toxicología Ambiental, Instituto de Investigaciones Biomédicas, Universidad Nacional Autónoma de México, Mexico City, Mexico; 5 Universidad de Guadalajara, Centro Universitario de Ciencias de la Salud, Departamento de Fisiología, Guadalajara, Jalisco, México; 6 Centro de Investigación en Salud Poblacional, Instituto Nacional de Salud Pública, Cuernavaca, Morelos, México

**Keywords:** cervical cancer, HPV persistence, human papillomavirus, human papillomavirus immune response, tumor microenvironment

## Abstract

Persistent infection with high-risk human papillomavirus (HR-HPV) is the primary cause of cervical cancer (CC). However, most HPV infections are transient and are cleared by effective host immune responses. Therefore, understanding the mechanisms that allow viral persistence and immune escape is critical to explaining why only a subset of women develops cervical lesions and invasive cancer. Although cervical cancer is largely preventable through vaccination and screening, it remains a major cause of cancer-related morbidity and mortality among women worldwide, with a disproportionate burden in low- and middle-income countries. This significant disparity underscores the ongoing inequities in access to HPV vaccination, screening, and timely treatment, which hinder the World Health Organization aim to eliminate cervical cancer as a public health concern by 2030. This narrative review examines how viral, host, and microenvironmental factors interact to promote HR-HPV persistence and cervical cancer development, with particular attention to evidence from Mexican women. HR-HPV can evade immune surveillance by interfering with innate immune recognition, interferon signaling, antigen presentation, and cytotoxic lymphocyte activity. These alterations are compounded by cervicovaginal dysbiosis, immunosuppressive cytokines, regulatory-cell expansion, host genetic variation, and altered microRNA expression. Together, these interconnected processes impair viral clearance and create conditions that favor the progression of cervical lesions to invasive cancer. A comprehensive understanding of these mechanisms may facilitate the identification of biomarkers of HPV persistence and disease progression, while supporting the development of more effective immunotherapeutic strategies and precision prevention approaches, particularly for populations disproportionately affected by cervical cancer.

## 1 Literature search strategy

A structured literature search was conducted in PubMed between August 2025 and August 2026 to identify scientific literature addressing the molecular and immunological mechanisms underlying high-risk human papillomavirus (HR-HPV) persistence and cervical carcinogenesis. Search terms included combinations of “human papillomavirus,” “HPV,” or “high-risk HPV” with terms related to viral persistence and carcinogenesis, viral immune evasion and HPV proteins, innate and adaptive immunity, cytokines and immune checkpoints, cervicovaginal microbiota, host genetic, microRNA-related factors, public health, vaccination, and therapeutics.

Only English-language scientific publications were considered, including original research articles, systematic reviews, meta-analyses, and relevant narrative reviews. Seminal studies were retained when necessary to describe established molecular and immunological mechanisms, while recent evidence was incorporated to reflect current advances. The scientific literature search was complemented by official reports and publicly available resources from international health and cancer surveillance organizations to obtain epidemiological data and information on HPV vaccination and cervical cancer prevention and elimination strategies. Studies and institutional sources were selected according to their relevance to the scope of the review. As this was a narrative review, no formal risk-of-bias assessment or quantitative evidence synthesis was performed.

## 2 Introduction

Cervical cancer (CC) remains a significant global health issue, with approximately 600,000 new cases and 280,000 deaths estimated worldwide in 2024 (1). More than 200 types of human papillomavirus (HPV) have been identified and are classified according to their oncogenic potential. Low-risk HPV types are primarily associated with benign lesions, such as anogenital warts, whereas high-risk HPV (HR-HPV) types are associated with cervical intraepithelial lesions and cervical cancer (2). Among the carcinogenic HPV types, HPV16 and HPV18 are particularly relevant, as they account for the majority of HPV-positive invasive cervical cancers worldwide (3–5). Although HPV infection is common among sexually active individuals, most newly acquired infections are transient and resolve spontaneously. In contrast, persistent HR-HPV infection increases the risk of developing cervical intraepithelial lesions and, eventually, cervical cancer (6–8).

The prevalence of HPV infection differs by age and geographic region. It is generally higher among younger women, with a first peak before 25 years of age, although age-specific patterns vary across populations (9). Despite the widespread implementation of HPV vaccination and cervical cancer screening programs, the disease continues to pose a major public health challenge, particularly in low- and middle-income countries, where access to prevention and treatment remains limited (10). Women living with human immunodeficiency virus (HIV) are also at substantially greater risk of developing cervical cancer (11). These differences in age, geographic setting, immune status, and access to preventive healthcare are important considerations for understanding why some HPV infections persist and progress toward cervical cancer.

The outcome of HPV infection is largely determined by the dynamic interplay between viral immune evasion strategies and the host immune response. HR-HPV has evolved sophisticated mechanisms to evade both innate and adaptive immunity, primarily through the activities of the early proteins E5, E6, and E7, which interfere with pathogen recognition receptor signaling, type I interferon responses, antigen processing and presentation, and immune cell activation (12). Persistent HR-HPV infection progressively remodels the local immune microenvironment, promoting immune suppression and creating conditions that favor viral persistence, lesion progression, and malignant transformation (12, 13). Among HR-HPV-positive women, high parity, long-term oral contraceptive use, smoking, and coinfection with other sexually transmitted agents have been identified as potential cofactors associated with progression to High-Grade Squamous Intraepithelial Lesion (HSIL) and invasive cervical cancer (14). For instance, a cross-sectional study found a higher prevalence of active herpes simplex virus type 2 (HSV-2) infection among HR-HPV-positive than HR-HPV-negative women (15), while pooled case–control evidence suggests that HSV-2 may act in conjunction with HPV as a cofactor associated with an increased risk of invasive cervical carcinoma (16).

This review provides a comprehensive overview of the mechanisms by which HR-HPV evades immune surveillance during cervical carcinogenesis. We discuss recent advances in our understanding of antiviral immunosuppression, host genetic susceptibility, the tumor immune microenvironment, highlighting their implications for cervical cancer prevention and treatment.

## 3 Natural history of HPV infection and cervical carcinogenesis

### 3.1 Natural history of cervical HPV infection

CC develops through a well-characterized multistep process that begins with infection by HPV, one of the most common sexually transmitted pathogens worldwide. In most individuals, HPV infection is transient and asymptomatic, with the virus being cleared spontaneously within 1–2 years. However, infections with HR-HPV genotypes, particularly HPV16 and HPV18, can persist in a subset of individuals (17).

The unique HPV life cycle contributes to the clinically silent nature of the infection. Viral replication is tightly linked to epithelial differentiation, and newly formed virions are released through the normal desquamation of superficial epithelial cells rather than through cell lysis. As a result, HPV infection causes little tissue damage and elicits only a limited inflammatory response, delaying immune detection and increasing the likelihood of persistent infection (18, 19).

Persistent HR-HPV infection is the key driver of disease progression, promoting the transition from low-grade squamous intraepithelial lesions [LSIL; corresponding to cervical intraepithelial neoplasia grade 1 (CIN1)] to high-grade squamous intraepithelial lesions (HSIL; corresponding to CIN2 and CIN3), and ultimately to invasive cervical carcinoma (20). This process is mediated by sustained viral oncogene expression, innate and adaptive immune evasion, and the progressive accumulation of genetic and epigenetic alterations in host cells. Environmental and infectious cofactors may further modify the risk of progression.

### 3.2 Host immune responses to HPV infection

Epithelial cells constitute the first line of innate immune defense against HPV by detecting viral components through pattern recognition receptors (PRRs) expressed on the cell surface and within intracellular compartments. During infection, viral nucleic acids function as pathogen-associated molecular patterns (PAMPs) that are recognized by several endosomal toll-like receptors (TLRs). Among these, TLR3 detects double-stranded RNA (dsRNA), TLR7 and TLR8 recognize single-stranded RNA (ssRNA), and TLR9 senses unmethylated CpG-rich DNA (21).

Recognition of these viral ligands initiates intracellular signaling cascades that coordinate the antiviral response. TLR3 signals through the adaptor protein TIR-domain-containing adaptor-inducing interferon-β (TRIF), whereas TLR7, TLR8, and TLR9 primarily signal via myeloid differentiation primary response protein 88 (MyD88). These pathways activate downstream kinases and transcription factors, including nuclear factor-κB (NF-κB) and interferon (IFN) regulatory factors (IRFs), resulting in the expression of inflammatory cytokines and antiviral mediators (22).

In the cytosol, retinoic acid-inducible gene I (RIG-I)-like receptors (RLRs), particularly RIG-I and melanoma differentiation-associated protein 5 (MDA5), recognize distinct forms of viral RNA, whereas cyclic GMP-AMP synthase (cGAS) detects cytosolic double-stranded DNA. Upon RNA recognition, RIG-I and MDA5 signal through the adaptor mitochondrial antiviral-signaling protein (MAVS), promoting the activation of TANK-binding kinase 1 (TBK1) and IκB kinase ε (IKKε), which subsequently activate interferon regulatory factors 3 and 7 (IRF3/IRF7). In parallel, DNA binding activates cGAS to synthesize cyclic GMP-AMP (cGAMP), which binds to and activates stimulator of interferon genes (STING), leading to TBK1-mediated activation of IRF3 (21, 23). Together, these signaling pathways drive the production of IFN-I, primarily IFN-α and IFN-β, which act in both infected and neighboring cells through the interferon-α/β receptor (IFNAR). Activation of IFNAR stimulates the Janus kinase/signal transducer and activator of transcription (JAK–STAT) pathway, promoting the formation of the interferon-stimulated gene factor 3 (ISGF3) complex, composed of STAT1, STAT2, and IRF9. ISGF3 subsequently induces the expression of hundreds of interferon-stimulated genes (ISGs), establishing a robust antiviral state that restricts viral replication and limits the spread of infection (24, 25).

Effective HPV clearance also requires a robust cell-mediated adaptive immune response. This response is characterized by the production of pro-inflammatory cytokines, including tumor necrosis factor-α (TNF-α) and T helper 1 (Th1)-associated cytokines, particularly IFN-γ. These cytokines promote cytotoxic T-cell activation, enhance antiviral activity, and support the development of a robust adaptive immune response (26–29).

### 3.3 Viral persistence and failure of immune clearance

Viral persistence occurs when innate and adaptive immune responses fail to eliminate HPV-infected epithelial cells. The non-lytic HPV life cycle contributes to this outcome by causing minimal tissue damage and limited inflammation. This weak initial inflammatory response can delay immune-cell recruitment and the development of effective adaptive immunity.

In addition, persistent HPV infection and lesion progression are characterized by impaired immune surveillance and the establishment of an immunosuppressive tumor microenvironment, marked by Th2- and Th17-skewed immune responses, enrichment of M2-like macrophages and regulatory T cells (Tregs), and increased production of immunosuppressive cytokines (30–33). This immunological shift suppresses antiviral immunity and creates a microenvironment that supports chronic inflammation, uncontrolled cell proliferation, angiogenesis, tissue invasion, and metastasis (34–37).

## 4 HPV-mediated evasion of innate antiviral immunity

HPV strengthens this immunosuppressive environment through the actions of its early proteins (E1, E2, E4, E5, E6, and E7), playing central roles in establishing persistent infection by suppressing antiviral immunity from the earliest stages of viral replication (Figure 1). Together, these proteins interfere with innate immune signaling, alter host gene expression, and reshape the local immune microenvironment, thereby creating conditions that favor viral persistence and disease progression (12).

**Figure 1:**
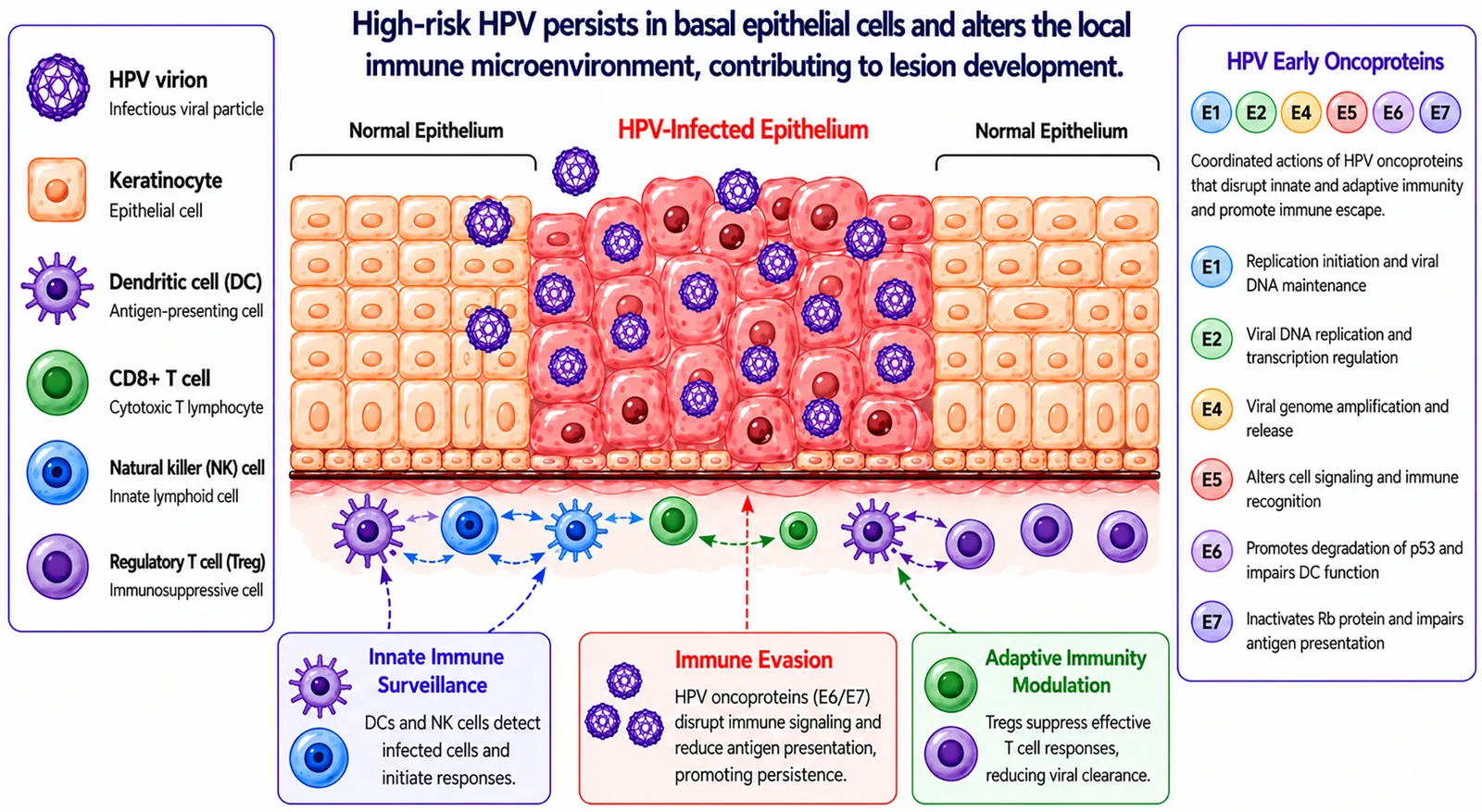
Schematic representation of the immunological events associated with persistent high-risk human papillomavirus (HR-HPV) infection in the cervical epithelium. Persistent HR-HPV infection promotes immune evasion by altering both innate and adaptive immune responses. HPV-infected keratinocytes interact with dendritic cells (DCs), natural killer (NK) cells, CD8⁺ T lymphocytes, and regulatory T (Treg) cells within the local immune microenvironment. Viral oncoproteins, particularly E6 and E7, interfere with antigen presentation, innate immune signaling, and cytotoxic immune responses, facilitating viral persistence and lesion progression. The figure also summarizes the principal functions of the HPV early proteins (E1–E7) during viral replication and host immune modulation. This figure was created with the assistance of generative artificial intelligence **(**OpenAI ChatGPT/DALL·E). The scientific content, interpretation, and final design were reviewed and approved by the authors.

Among them, the high-risk HPV oncoproteins E6 and E7 are the principal mediators of immune dysregulation and malignant transformation (38). Their sustained expression, which frequently follows viral genome integration into the host genome, not only disrupts cell-cycle regulation but also profoundly influences the composition and function of the tumor microenvironment. Through E6-mediated degradation of p53 and E7-mediated inactivation of pRb (39–42), these oncoproteins interfere with multiple signaling pathways involved in both innate and adaptive immunity, including NF-κB, interferon regulatory factors 1 and 3 (IRF1, IRF3), and JAK/STAT signaling (43–45).

Although E6 and E7 account for many of the oncogenic and immunomodulatory effects of HR-HPV, the other early proteins also make important contributions to viral persistence. E1, E2, E4, and E5 target multiple components of the host antiviral response, including pathogen-sensing and IFN signaling pathways, thereby facilitating viral replication, immune escape, and lesion progression.

### 4.1 Disruption of pattern-recognition receptor signaling by HPV early proteins

PRRs constitute the first line of defense against HPV infection by detecting viral nucleic acids and initiating IFN-I responses. To establish persistent infection, HPV has evolved multiple mechanisms to suppress these pathways through the coordinated actions of its early proteins. Collectively, E1, E2, E4, E5, E6, and E7 interfere with TLR-dependent signaling as well as cytosolic RNA- and DNA-sensing pathways, thereby limiting viral recognition, IFN production, and the induction of ISGs.

The HPV E1 protein, best known for its essential roles in viral DNA replication and cell-cycle regulation, also functions as an important modulator of innate immunity. Initial evidence from HPV18-positive HeLa cells showed that E1 silencing altered the expression of genes involved in TLR and IFN signaling, interferon-stimulated gene (ISG) responses, and apoptosis (46). Consistent with these findings, E1 proteins from both high-risk (HPV16 and HPV18) and low-risk (HPV11) types suppress the expression of IFNB1, IFNL1, C-C motif chemokine ligand 5 (CCL5), and radical S-adenosyl methionine domain containing 2 (RSAD2), indicating that immune modulation is conserved across HPV genotypes (47). Similar antiviral suppression has also been reported for HPV8 E1, which inhibits melanoma differentiation-associated protein 5 (MDA5)-mediated induction of IFN-I and interleukin-6 (IL-6), suggesting that this function extends to both mucosal and cutaneous HPVs (48). More recently, HPV16 E1 was shown to broadly inhibit IFN-I and ISG responses triggered by RNA viruses, DNA viruses, and synthetic viral RNA analogs through interactions with multiple components of innate immune signaling, includingRIG-I, MDA5, mitochondrial antiviral signaling protein (MAVS), TIR domain-containing adaptor molecule 1 (TRIF), stimulator of interferon response cGAMP interactor 1 (STING), and signal transducer and activator of transcription 1 (STAT1) (49). By disrupting both IRF3 activation and JAK–STAT signaling, E1 suppresses interferon production as well as downstream antiviral gene expression.

The E2 protein also contributes to the disruption of viral sensing. In addition to cooperating with E1 during viral replication through binding to the long control region and facilitating origin recognition, the E2 protein also contributes to immune modulation. During episomal infection, E2 represses E6 and E7 transcription, thereby maintaining controlled host-cell proliferation (50). Beyond this regulatory role, E2 suppresses several innate immune pathways, including STING- and IFN-κ-dependent signaling, and its expression has been associated with reduced STING levels in low-grade cervical lesions (51). Consistent with these observations, HPV16 E2 inhibits the expression of IFN-β, ISGs, and NF-κB-dependent inflammatory genes by targeting multiple antiviral signaling pathways, including RIG-I/MDA5, MAVS, TLR3–TRIF, and cGAS–STING (52).

The E4 protein, expressed from the spliced E1^E4 transcript, is highly abundant in differentiated epithelial cells, where it plays an essential role in virion maturation and release (53). In addition to facilitating viral egress through interactions with the keratin network, HPV16 E4 contributes to G2-phase cell-cycle arrest and modulates of mitogen-activated protein kinase (MAPK) signaling (54). Moreover, E4 has also been shown to suppress virus-induced type I IFN and ISG expression, further highlighting its contribution to immune evasion during the productive stage of the viral life cycle (55).

The HPV16 E5 protein complements the immunomodulatory activities of the viral early expressed proteins by targeting both, antiviral signaling and antigen presentation. E5 suppresses basal IFN-κ expression, thereby attenuating JAK–STAT-dependent induction of ISGs. At the same time, it interferes with antigen processing and major histocompatibility complex (MHC) class I-mediated antigen presentation, reducing immune recognition of infected cells and facilitating viral persistence (56–58).

Among the HPV proteins, however, E6 and E7 function as the principal regulators of PRR signaling, targeting multiple RNA- and DNA-sensing pathways. HPV16 E6 suppresses RIG-I-mediated innate immune signaling by interacting with TRIM25 and its regulator USP15, enhancing K48-linked ubiquitination and degradation of TRIM25. This impairs TRIM25-mediated K63-linked ubiquitination of RIG-I and its subsequent interaction with MAVS, thereby attenuating downstream antiviral signaling (59). Furthermore, E6 inhibits RIG-I–MAVS signaling, suppresses IFN-β promoter activation, reduces STAT1 phosphorylation, and decreases the expression of antiviral cytokines and interferon-stimulated genes, including IFNB1, CCL5, CCL20, MX1, ISG15, and OAS1 (59).

HPV16 and HPV18 E7 interfere with cytosolic DNA sensing through distinct mechanisms targeting the cGAS–STING pathway. HPV18 E7 directly interacts with STING, selectively suppressing STING-dependent NF-κB activation and attenuating downstream innate immune responses (60), whereas studies in HPV16-positive head and neck squamous cell carcinoma (HNSCC) models have shown that HPV16 E7 promotes NLRX1-dependent autophagic degradation of STING, resulting in diminished type I IFN responses (61). In addition, HPV16 and HPV18 E7 induce the H3K9 methyltransferase SUV39H1, which mediates epigenetic silencing of the viral nucleic acid sensors cGAS and RIG-I as well as the adaptor STING through H3K9 trimethylation at their promoter regions (62).

Regarding the TLRs, TLR9 is one of the best characterized targets of HPV-mediated immune evasion. TLR9 recognizes viral double-stranded DNA within endosomal compartments and initiates type I IFN responses (63). HPV16 E7 represses TLR9 transcription by recruiting a repressive complex composed of NF-κB p50/p65, estrogen receptor-α, HDAC1, and JARID1B to the *TLR9* promoter, leading to epigenetic silencing and impaired IFN-I production (64, 65). Interestingly, this suppression appears to be transient during the early stages of infection, with TLR9 expression returning to baseline within 24 h (66). A related mechanism has also been described for HPV38, in which E6 and E7 promote the accumulation of ΔNp73α, resulting in the recruitment of DNMT1 and EZH2 to the *TLR*9 promoter and subsequent H3K27 trimethylation (67). Restoration of *TLR9* expression inhibits the proliferation of HPV38 E6/E7-expressing cells, suggesting that *TLR9* repression represents a conserved immune evasion mechanism shared by multiple HPV types.

Clinical observations, indicate that *TLR9* expression is highly dynamic during HPV infection and cervical carcinogenesis. Women who successfully clear HPV16 infection exhibit higher TLR9 expression, whereas reduced expression is associated with short-term viral persistence (68). In contrast, persistent HR-HPV infection and progression to CIN and cervical cancer are frequently accompanied by increased TLR9 expression in cervical tissues across diverse patient populations (69–73). Likewise, elevated TLR9 expression in peripheral blood mononuclear cells (PBMCs) and increased circulating TLR9 levels have been reported in women with cervical lesions and cervical cancer, highlighting its potential value as a biomarker of disease progression (74). These findings suggest that, during the early stages, HPV transiently suppresses TLR9 signaling to evade innate immune recognition and facilitate viral persistence. However, as the infection becomes chronic and neoplastic lesions develop, TLR9 expression increases, likely reflecting sustained exposure to viral DNA and remodeling of the tumor microenvironment rather than restoration of an effective antiviral response (63).

These mechanisms establish a sustained suppression of innate immune signaling, thereby facilitating viral persistence and immune evasion. Figure 2 summarizes the major PRR-mediated pathways involved in viral DNA and RNA sensing, as well as the mechanisms by which HPV proteins interfere with these pathways to inhibit type I interferon production and the establishment of an antiviral state**.**

**Figure 2:**
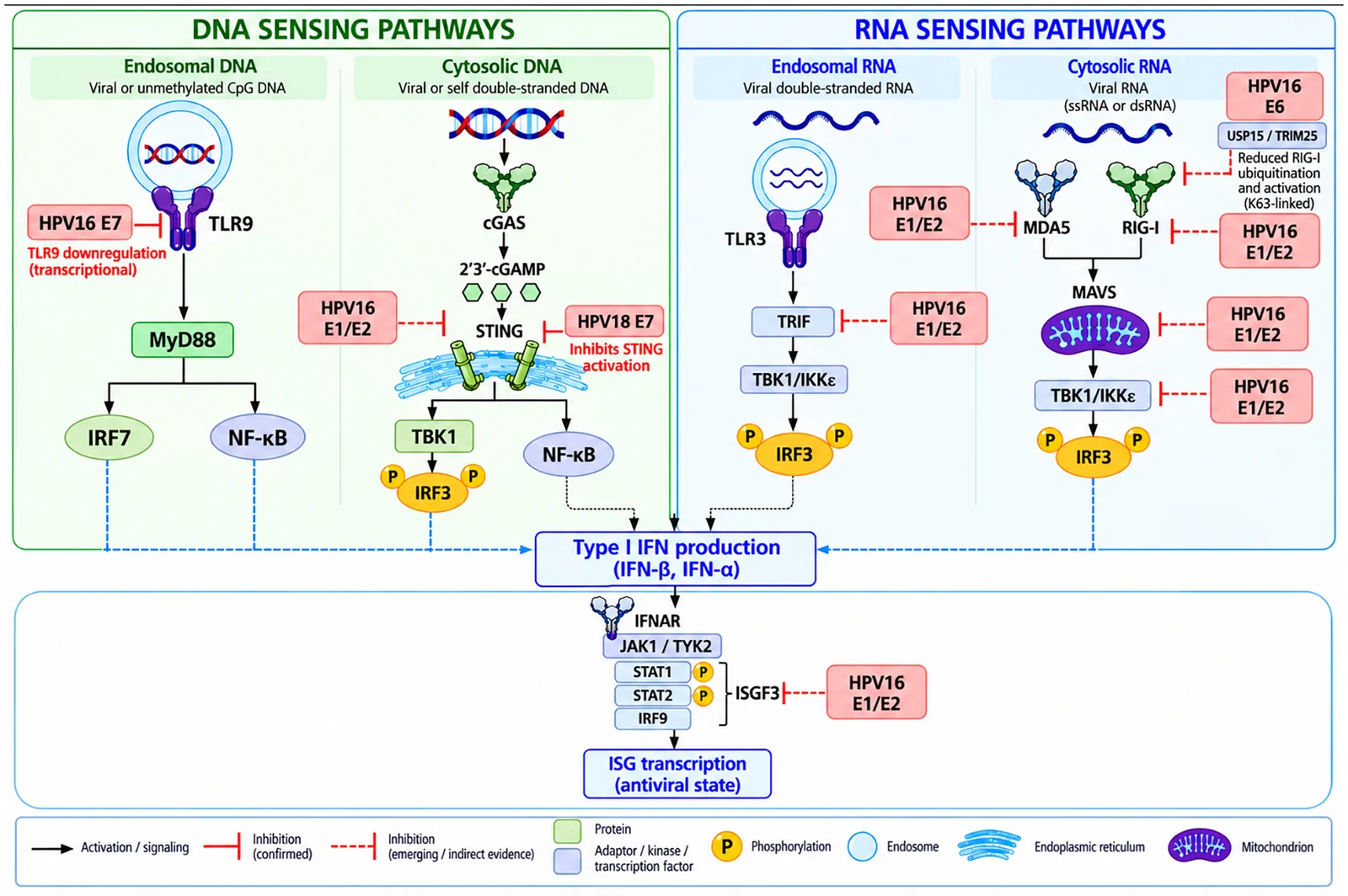
HPV early proteins suppress innate immune recognition. High-risk HPV early proteins inhibit DNA- and RNA-sensing pathways by targeting TLR9, cGAS–STING, TLR3–TRIF, RIG-I/MDA5–MAVS, and downstream TBK1–IRF3 and JAK–STAT signaling. These coordinated activities reduce type I interferon (IFN-α/β) production and interferon-stimulated gene (ISG) expression, thereby weakening antiviral responses and promoting immune evasion and viral persistence. This figure was created with the assistance of generative artificial intelligence **(**OpenAI ChatGPT/DALL·E). The scientific content, interpretation, and final design were reviewed and approved by the authors.

## 5 The cervicovaginal microenvironment in HPV persistence

In addition to the direct viral mechanisms of immune evasion, the outcome of HPV infection is influenced by the local cervicovaginal microenvironment. In particular, the composition of the cervicovaginal microbiota can affect epithelial barrier integrity, inflammatory signaling, and local immune homeostasis. Alterations in these microbial communities may therefore create conditions that favor HPV persistence and contribute to the transition from antiviral immune activation to chronic inflammation and immunosuppression.

### 5.1 Cervicovaginal microbiome and local immune homeostasis

The bidirectional relationship between the immune system and the cervicovaginal microbiota has been widely investigated in both, observational and experimental studies. However, the mechanisms through which host immune responses and cervicovaginal microbial communities interact and influence one another remain incompletely understood (75).

The cervicovaginal microbiota of reproductive-age women exhibits relatively low microbial diversity compared to that of other body sites and is commonly classified into five community-state types (CSTs) (76). Four CSTs are dominated by Lactobacillus species [*L. crispatus* (CST-I), *L. gasseri* (CST-II), *L. iners* (CST-III), and *L. jensenii* (CST-V)], whereas CST-IV is characterized by a depletion of lactobacilli and enrichment of diverse anaerobic bacteria, including *Gardnerella* spp., *Fannyhessea vaginae* (formerly *Atopobium vaginae*), *Prevotella*, *Sneathia*, *Megasphaera*, and *Mobiluncus*, taxa consistently associated with vaginal dysbiosis and persistent high-risk HPV infection (77–79). While *L. crispatus*-dominated communities are considered a hallmark of cervicovaginal health, CST-IV is associated with a pro-inflammatory microenvironment that disrupts local immune homeostasis and may favor HPV persistence and disease progression (77).

### 5.2 Microbiome dysbiosis and persistent HPV infection

Cervicovaginal dysbiosis is increasingly recognized as a potential cofactor in HPV persistence and the development of CIN (75, 80, 81). In particular, *L. crispatus* is consistently associated with HPV clearance and a reduced risk of viral persistence, whereas *L. iners* and dysbiotic communities enriched with *Gardnerella vaginalis*, *Atopobium vaginae*, *Prevotella*, *Megasphaera*, and *Sneathia spp*. have been associated with persistent HPV infection, CIN, and cervical cancer (Figure 3) (80, 82, 83).

**Figure 3:**
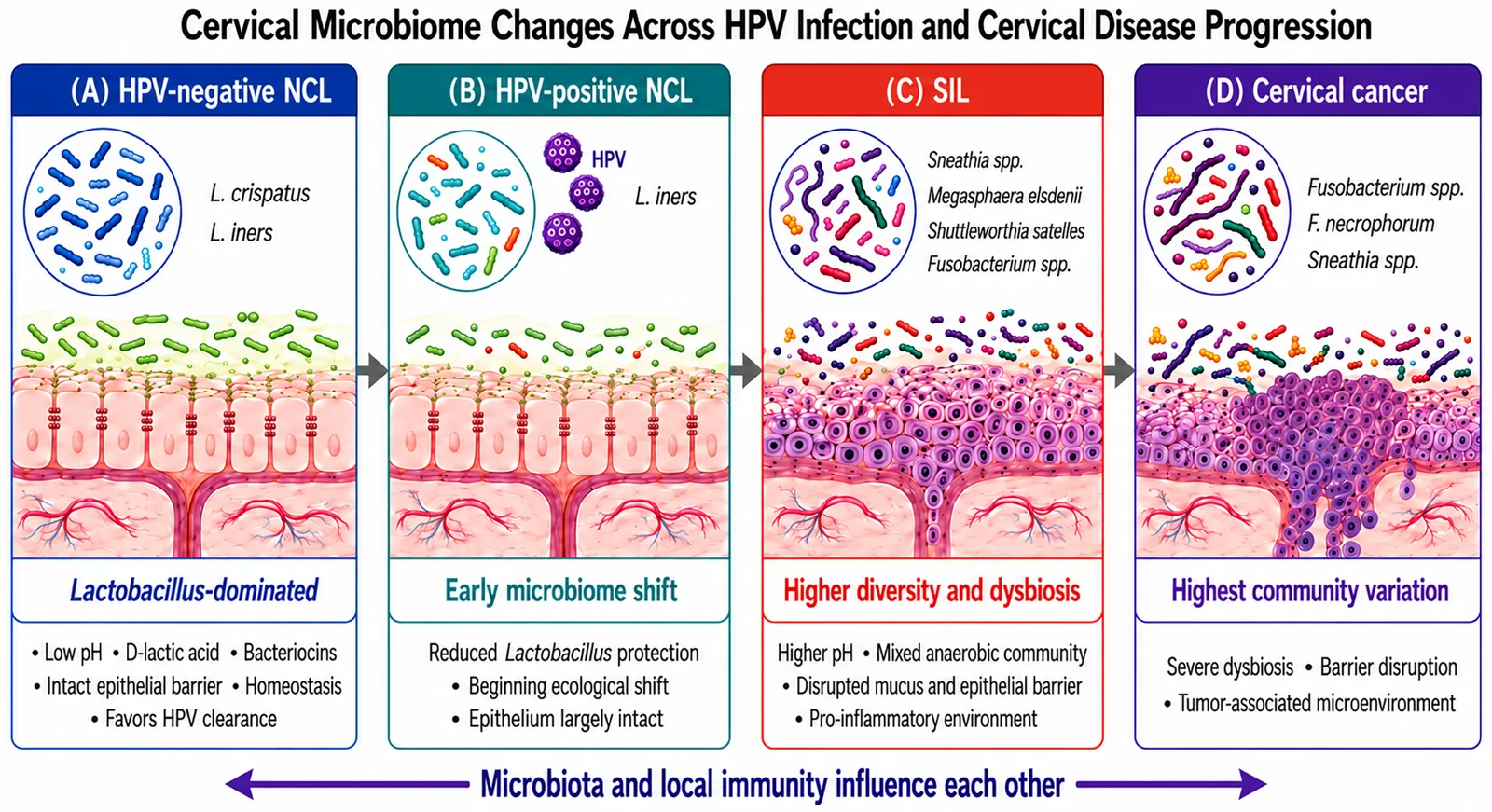
Cervical microbiome changes during HPV infection and cervical disease progression. Schematic representation of the cervical microbiota across disease stages. **(A)** HPV-negative normal cervical lining (NCL) is dominated by *Lactobacillus* spp., supporting a low-pH, homeostatic environment that favors HPV clearance. **(B)** HPV-positive NCL shows an early microbial shift with reduced *Lactobacillus* predominance while the epithelial barrier remains largely intact. **(C)** Squamous intraepithelial lesions (SIL) are characterized by increased microbial diversity, anaerobic dysbiosis, epithelial barrier disruption, and a pro-inflammatory microenvironment. **(D)** Cervical cancer exhibits severe dysbiosis, high microbial community variation, and a tumor-associated microenvironment. This figure was created with the assistance of artificial intelligence (OpenAI ChatGPT/DALL·E) and subsequently reviewed and edited by the authors for scientific accuracy.

Several mechanisms have been proposed to explain how cervicovaginal dysbiosis contributes to HPV persistence and disease progression. Microbe-associated molecular patterns (MAMPs), including bacterial lipoproteins, peptidoglycan, and lipoteichoic acid, together with virulence factors produced by dysbiosis-associated bacteria such as *Gardnerella*, activate PRRs, particularly TLR2, on cervical epithelial cells and antigen-presenting cells, resulting in NF-κB activation and increased production of cytokines such as IL-6, IL-8, TNF-α, and other inflammatory mediators (75, 84–87). Consistent with these findings, greater cervicovaginal bacterial diversity has been associated with higher concentrations of proinflammatory cytokines in the female genital tract (85). In parallel, bacterial sialidases and other hydrolytic enzymes degrade cervicovaginal mucins, compromising the mucus barrier, disrupting epithelial integrity, and increasing epithelial permeability, thereby facilitating HPV access to basal epithelial cells (86, 88, 89). Through these effects on the mucus barrier, epithelial integrity, and local inflammatory responses, dysbiosis may create conditions that favor persistent HPV infection and increase the risk of SIL (90).

Beyond its contribution to HPV persistence, cervicovaginal dysbiosis has also been implicated in remodeling the cytokine profile of the cervical microenvironment during neoplastic progression, promoting an immunosuppressive milieu that supports lesion progression and tumorigenesis.

In a pilot study, Audirac-Chalifour et al. reported that *Fusobacterium*-dominated microbial communities were associated with increased cervical expression of IL-4 and Transforming growth factor-β1 (TGF-β1), suggesting a shift toward Th2-polarized immune responses (Figure 1) (91). Dysbiosis-associated bacteria, particularly *Fusobacterium* and *Sneathia*, have also been implicated in epithelial dysfunction and chronic inflammation. *Fusobacterium* has been shown to disrupt E-cadherin/β-catenin signaling and activate NF-κB in epithelial cells (92, 93), whereas *Sneathia* is consistently associated with epithelial barrier disruption, inflammatory responses, and persistent HPV infection (83, 94), although the molecular mechanisms underlying these effects remain to be fully elucidated.

Similar associations among cervical dysbiosis, chronic inflammation, and cervical neoplasia have been reported across geographically and ethnically diverse populations, highlighting the need for longitudinal and mechanistic studies to determine how microbial alterations contribute to the transition from persistent HPV infection to an immunosuppressive and tumor-promoting microenvironment (95–97).

## 6 Tumor microenvironment and immunosuppressive regulation

Several studies have shown that HPV-infected cells exhibit reduced expression of antiviral IFNs and immune-stimulatory cytokines, accompanied by increased production of immunosuppressive and pro-tumorigenic mediators such as IL-10, TGF-β1, IL-4, IL-6, and vascular endothelial growth factor (VEGF) (30, 34, 98–100). In parallel, E6 and E7 suppress the expression of chemokines required for the recruitment and activation of dendritic cells, natural killer (NK) cells, and effector T lymphocytes, thereby limiting immune cell infiltration and promoting an environment that supports angiogenesis, tumor progression, and persistent infection (101, 102).

### 6.1 Immunosuppressive cytokines IL-10 and TGF-β1 in the tumor microenvironment

IL-10 and TGF-β1 are central mediators of immunosuppression during persistent HPV infection and cervical carcinogenesis. High-risk HPV actively contributes to this immunosuppressive cytokine milieu by directly regulating host gene expression. HPV16 E6 and E7 increase TGF-β1 promoter activity in cervical cancer cells, whereas HPV16 E2 binds to regulatory elements within the IL-10 promoter and enhances IL-10 transcription (99, 103). In turn, IL-10 has been shown to enhance HPV16 early promoter activity through STAT3-dependent regulation of the viral upstream regulatory region, potentially establishing a positive feedback loop that reinforces viral oncogene expression, immune tolerance, and viral persistence (104).

IL-10 suppresses both innate and adaptive immune responses by impairing antigen presentation and T-cell activation. Mechanistically, it reduces the expression of MHC class II molecules and the co-stimulatory proteins CD80 and CD86 on antigen-presenting cells, thereby limiting their ability to activate effector T cells (105). Consistent with these immunomodulatory effects, elevated IL-10 expression is associated with persistent HPV infection, cervical lesion progression, and impaired cell-mediated immunity, supporting a potential role in immune evasion during cervical carcinogenesis (36, 106).

TGF-β1 exerts similarly broad immunosuppressive effects but also plays a context-dependent role in tumor development. Under physiological conditions, TGF-β1 signaling contributes to tissue homeostasis by regulating epithelial cell proliferation, differentiation, and apoptosis. However, as cervical lesions progress, this pathway is frequently co-opted to support tumor growth and immune evasion (107).

In established cervical tumors, TGF-β1 suppresses NK cell cytotoxicity, promotes the accumulation and functional stability of Tregs, and further weakens antitumor immune responses. Accordingly, increased expression of TGF-β1 and its receptors has been documented in cervical lesions and invasive cervical cancer, indicating sustained activation of this pathway throughout disease progression (108–110).

### 6.2 Regulatory T cells in cervical cancer

Tregs cells, characterized by the CD4⁺CD25⁺high FoxP3⁺ phenotype, are major mediators of immunosuppression in cervical cancer (111–113). Increased frequencies of Tregs have consistently been reported in both the peripheral blood and tumor tissues of women with cervical cancer compared with healthy controls, highlighting their role in establishing and maintaining an immunosuppressive tumor microenvironment (111, 114, 115).

The accumulation of Tregs increases with disease progression and is associated with advanced-stage cervical cancer, impaired antitumor immunity, and poor clinical outcomes. In addition, disruption of the Th17/Treg balance correlates with disease stage and immune dysregulation, indicating that an imbalance between pro-inflammatory and immunosuppressive T-cell subsets contributes to cervical carcinogenesis and immune escape (116, 117). Consistent with these observations, elevated circulating Treg frequencies have been identified as a characteristic feature of cervical cancer (115).

Tregs not only directly suppress effector T-cell function but also actively shape the tumor microenvironment through the secretion of IL-10 and TGF-β1. These immunosuppressive cytokines inhibit antigen presentation and cytotoxic immune responses while promoting the induction, expansion, and functional stability of additional FoxP3⁺ Tregs, thereby establishing a self-perpetuating regulatory network that contributes to tumor immune escape (118–120).

### 6.3 Metabolic immunosuppression through the CD39–CD73–adenosine axis

Beyond its direct immunoregulatory functions, TGF-β1 may also link cytokine-mediated immunosuppression to metabolic immune escape by inducing the ectonucleotidases CD39 and CD73. These enzymes catalyze the generation of extracellular adenosine, a potent immunosuppressive metabolite produced by cervical cancer cells and released in extracellular vesicles, thereby further reinforcing the inhibitory tumor microenvironment (121, 122).

In addition to cytokine-mediated immunosuppression, cervical tumors exploit metabolic pathways to evade antitumor immunity responses. One of the best-characterized mechanisms is the accumulation of extracellular adenosine within the tumor microenvironment. Under conditions of chronic inflammation, hypoxia, and tissue damage, extracellular ATP released by tumor and stromal cells is sequentially hydrolyzed by the ectonucleotidases CD39 and CD73, resulting in the generation of adenosine. This immunosuppressive metabolite primarily exerts its effects through the activation of the A2A and A2B adenosine receptors expressed on immune cells (123–125).

Adenosine signaling broadly suppresses antitumor immune responses. Activation of A2A and A2B receptors inhibits T-cell proliferation, cytokine production, and cytotoxic function while impairing the maturation and effector activity of NK cells. At the same time, adenosine enhances the suppressive activity of Tregs, tumor-associated macrophages, and myeloid-derived suppressor cells (MDSCs), thereby reinforcing immune tolerance within the cervical tumor microenvironment (123, 124). In addition, adenosine stimulates the production of immunosuppressive cytokines, including IL-10, providing a mechanistic link between metabolic reprogramming and cytokine-mediated immune suppression (126).

Growing evidence indicates that this pathway is actively involved in cervical cancer. Mesenchymal stromal cells isolated from cervical tumors express high levels of CD39 and CD73 and efficiently convert extracellular nucleotides into adenosine, thereby suppressing the cytotoxic activity of T lymphocytes (127). Moreover, these stromal cells enhance CD73 expression in cervical cancer cells through TGF-β1 signaling (121), whereas TGF-β1 promotes the release of extracellular vesicles enriched in CD39 and CD73, potentially amplifying adenosine production both locally and throughout the tumor microenvironment (122). Consistent with the relevance of this pathway, elevated CD73 and TGF-β1 expression in cervical cancer tissues is strongly associated with programmed death-ligand 1 (PD-L1) expression, increased frequencies of programmed death-1 (PD-1)⁺CD8⁺ T cells, and disease progression, highlighting the central role of adenosine-mediated immune suppression in cervical cancer progression (128).

## 7 Immune dysfunction during cervical cancer progression

7.1 T-cell dysfunction during cervical carcinogenesis: impaired activation, anergy, and exhaustion.

Under physiological conditions, HPV-derived antigens are processed and presented by MHC class I and II molecules, leading to the activation and recruitment of HPV-specific CD8⁺ cytotoxic T lymphocytes and CD4⁺ helper T cells, respectively (129–132). These adaptive immune responses are essential for viral clearance and the regression of HPV-associated lesions (27, 132–134). On the other hand, HPV-mediated impairment of antigen presentation, together with reduced expression of co-stimulatory molecules on antigen-presenting cells, compromises effective T-cell priming, thereby promoting viral persistence and progression to cervical neoplasia (27, 129, 135).

One of the earliest consequences of inadequate antigen presentation is T-cell anergy, a hyporesponsive state that develops when T cells TCR-mediated antigen recognition (signal 1) in the absence of adequate co-stimulatory signaling (signal 2), primarily through the interaction between CD28 and CD80/CD86 (136). Anergic T cells remain viable but exhibit markedly reduced proliferation, diminished IL-2 production, and impaired effector function upon subsequent antigen encounter (136). In HPV-associated lesions, reduced MHC expression, impaired co-stimulatory signaling, and the local production of immunosuppressive cytokines collectively impair effective T-cell priming and promote the establishment of T-cell anergy, thereby limiting the development of robust antiviral cellular immunity and favoring viral persistence (137, 138).

As HPV infection persists and cervical lesions progress, continuous exposure to viral and tumor antigens promotes a more profound state of T-cell dysfunction characterized by impaired TCR signaling. A central molecular alteration underlying this process is the reduced expression of the CD3ζ chain (CD247), a critical signaling component of the TCR/CD3 complex. Reduced CD3ζ expression has been consistently reported in both peripheral blood and tumor-infiltrating lymphocytes from patients with cervical cancer, where it has been associated with impaired T-cell proliferation, reduced production of IL-2 and IFN-γ, and diminished antitumor activity (139–143).

Persistent antigenic stimulation may gradually promote the transition from anergy to T-cell exhaustion, a distinct differentiation state that develops during chronic viral infection and cancer (144, 145). Exhausted T cells are characterized by a progressive loss of proliferative capacity, impaired cytotoxic activity, and reduced production of effector cytokines, while simultaneously acquiring sustained expression of multiple inhibitory immune checkpoint receptors, including PD-1, cytotoxic T-lymphocyte-associated protein 4 (CTLA-4), T-cell immunoglobulin and mucin domain-containing protein 3 (TIM-3), T-cell immunoreceptor with Ig and ITIM domains (TIGIT), and lymphocyte activation gene-3 (LAG-3) (146–150).

Changes in the abundance and spatial distribution of T-cell populations accompany this progressive remodeling of the cervical immune microenvironment. Flow cytometric and immunohistochemical analyses of tumor-infiltrating lymphocytes (TILs) have demonstrated CD4⁺ and CD8⁺ T cells within both epithelial and stromal compartments of cervical lesions. With disease progression, TILs become more prominent in the stromal compartment, with CD8⁺ T cells generally predominating over CD4⁺ T cells (143). The comparatively low representation of CD4⁺ T cells in cervical cancer may be influenced, at least in part, by tumor-derived immunosuppressive factors. Furthermore, cervical cancer cells have been reported to secrete TGF-β, which suppresses lymphocyte proliferation and can preferentially induce apoptosis in CD4⁺ rather than CD8⁺ lymphocytes (151). These observations are consistent with previous reports describing reduced CD4⁺ T-cell representation and a relative predominance of CD8⁺ T cells within the cervical tumor microenvironment (141, 152, 153).

However, the presence or even relative accumulation of CD8⁺ T cells within cervical lesions does not necessarily indicate effective antitumor immunity. Features of exhaustion have been identified in both tumor-infiltrating and peripheral CD8⁺ T cells in cervical cancer and have been associated with impaired HPV-specific immunity and unfavorable clinical outcomes (145, 147, 148).

This dysfunctional state is further reinforced by the immunosuppressive milieu of cervical tumors. In addition to TGF-β1 and IL-10, mediators such as indoleamine 2,3-dioxygenase 1 (IDO1) and prostaglandin E₂ (PGE₂) contribute to the suppression of T-cell responses. At the same time, the recruitment and expansion of Tregs, myeloid-derived suppressor cells (MDSCs), tumor-associated macrophages (TAMs), and tolerogenic dendritic cells establish additional layers of immune regulation. Together, these soluble and cellular mechanisms interfere with T-cell priming and effector function, sustain an immunosuppressive microenvironment, and reinforce dysfunctional states such as anergy and exhaustion, thereby facilitating immune evasion by HPV-transformed cells (154–158).

### 7.2 Disruption of the AKNA–CD40/CD40L costimulatory axis

CD40–CD40L interactions are fundamental for the development of effective cellular and humoral immune responses. Engagement of CD40 on antigen-presenting cells by CD40L expressed on activated CD4⁺ T cells promotes dendritic cell maturation, cytokine production, upregulation of co-stimulatory molecules, and the generation of antigen-specific T-cell responses (159). CD40L expression is tightly regulated and preferentially maintained in Th1 cells through IL-12-dependent signaling, thereby sustaining long-lasting cellular immunity (160). An important regulator of this pathway is the AT-hook transcription factor (AKNA), which binds to A/T-rich motifs within the promoters of CD40 and CD40L, enhancing the transcription of both genes (161).

HR-HPV disrupts this regulatory axis through the activity of the E6 oncoprotein. Manzo-Merino et al. demonstrated that HPV16 and HPV18 E6 interact with AKNA and promote its proteasomal degradation, resulting in reduced CD40 expression in HPV-positive cervical cancer cells (162). Conversely, E6 silencing or restoration of AKNA expression increased CD40 levels and enhanced IL-8 transcription, indicating that AKNA contributes to immune and inflammatory signaling in cervical cancer cells (162). Consistent with these experimental findings, AKNA expression is markedly reduced in cervical tumors, and low AKNA levels are associated with unfavorable clinical outcomes. Moreover, polymorphisms within the AKNA promoter have been linked to altered AKNA expression and an increased susceptibility to cervical cancer, further supporting a potential role for AKNA dysregulation in cervical carcinogenesis (163). These findings suggest that HPV-mediated suppression of AKNA may impair CD40-dependent immune signaling, contributing to immune evasion and tumor progression.

### 7.3 NK cell dysfunction and immune evasion in cervical cancer

NK cells are essential components of the innate immune response against virally infected and transformed cells (164). However, both their phenotype and cytotoxic function become progressively compromised during cervical carcinogenesis, with functional alterations evident from precancerous lesions through invasive cervical cancer (165, 166). As the disease advances, NK cells acquire an exhausted phenotype characterized by increased expression of inhibitory receptors, including PD-1, T-cell immunoreceptor with Ig and ITIM domains (TIGIT), and T-cell immunoglobulin and mucin domain-containing protein 3 (Tim-3) (149). This is accompanied by reduced perforin production and decreased surface expression of the activating receptors natural cytotoxicity triggering receptor 3 (NCR3; NKp30) and natural cytotoxicity triggering receptor 1 (NCR1; NKp46), and killer cell lectin-like receptor subfamily K member 1 (NKG2D), together with increased expression of inhibitory receptors such as CD158a, CD158b, and NKG2A, ultimately leading to diminished cytotoxic activity (165, 167, 168). Despite this dysfunction, NK cells retain expression of the co-stimulatory receptors OX40 (CD134), 4-1BB (CD137), and inducible T-cell co-stimulator (ICOS), suggesting that their antitumor activity may be at least partially restored through immunotherapeutic approaches (169).

Cervical tumors further impair NK cell function through the release of soluble immunomodulatory ligands. Soluble MHC class I polypeptide-related sequences A and B (MICA and MICB), shed by tumor cells, induce downregulation of NKG2D on both NK and T cells, thereby reducing their cytotoxic potential (170, 171). Likewise, expression of the NKp30 ligand B7-H6 increases during cervical cancer progression and correlates with disease stage, whereas soluble B7-H6 is detectable in the serum of affected patients, supporting its potential utility as a biomarker of disease progression. Building on these findings, recent studies have developed biosensor- and exosome-based platforms for the detection of soluble B7-H6 and immune checkpoint ligands associated with NK- and T-cell exhaustion, underscoring their promise for clinical diagnosis and disease monitoring (172, 173).

Beyond local tumor-mediated suppression, systemic factors may also contribute to NK cell dysfunction. Gut microbiota dysbiosis in patients with cervical cancer has been associated with increased expression of multiple inhibitory receptors on NK cells, including PD-1, TIM-3, TIGIT, lymphocyte activation gene 3 protein (LAG-3), B and T lymphocyte attenuator (BTLA), and NKG2A, and correlates with poorer clinical outcomes. These findings suggest that alterations in the intestinal microbiome may further promote immune exhaustion and contribute to cervical cancer progression (174).

### 7.4 Apoptotic resistance and tumor-cell survival

The Fas (CD95)/Fas ligand (FasL, CD95L) pathway represents an important mechanism of immune evasion in CC. Cervical cancer cells frequently express FasL while remaining resistant to Fas-mediated apoptosis, enabling them to induce apoptosis in Fas-sensitive lymphocytes without undergoing cell death themselves (175). Consequently, tumor-reactive lymphocytes may be selectively eliminated, weakening local antitumor immunity and facilitating immune escape. Consistent with this mechanism, studies in HPV^+^ cell lines HeLa, SiHa and CaSki, have demonstrated dysregulation of the Fas/FasL system, whereas clinical studies have reported progressive loss of Fas expression accompanied by increased FasL expression during cervical carcinogenesis (176–181).

In addition to disrupting death receptor signaling, cervical tumors exploit immune checkpoint pathways to suppress antitumor immunity. Among these, the PD-1/PD-L1 axis is one of the best-characterized mechanisms of immunosuppression in CC. HPV oncoproteins E5, E6, and E7 can enhance PD-L1 expression through multiple oncogenic and inflammatory signaling pathways. Accordingly, elevated PD-L1 expression has been detected in CC cells, high-grade cervical lesions, and tumor-infiltrating immune cells, where it is associated with disease progression and poor clinical outcomes (182, 183).

Recent evidence has identified CKLF-like MARVEL transmembrane domain-containing protein 6 (CMTM6) as a critical regulator of PD-L1 stability. By preventing lysosomal degradation and promoting recycling of PD-L1 to the cell surface, CMTM6 sustains immune checkpoint signaling. Elevated levels of cellular, soluble, and exosome-associated CMTM6 have been reported in CC, suggesting that this protein reinforces PD-L1-mediated immunosuppression both within the tumor microenvironment and systemically (184).

These immune evasion mechanisms are reflected in the phenotype of tumor-infiltrating T cells. Patients with CC exhibit increased frequencies of CD4⁺CD28⁻ T lymphocytes, which have a reduced capacity to receive co-stimulatory signals through the CD80/CD86–CD28 pathway. In parallel, tumor-infiltrating CD8⁺ T cells frequently display an exhausted phenotype characterized by PD-1 expression and the co-expression of TIGIT, TIM-3, LAG-3, and NKG2A. This dysfunctional state becomes progressively more pronounced with disease advancement and is particularly evident in locally advanced tumors, where TIGIT expression is significantly increased (150).

## 8 Host cytokine genetic factors influencing HPV persistence

Host genetic variability is an important determinant of susceptibility to persistent HPV infection and CC. Among the most extensively investigated genetic factors are polymorphisms in cytokine genes, which may influence the balance between antiviral immunity and immunosuppressive responses. In Mexican women, the *IL-10* rs1800872 (−592 C > A) variant has been associated with an increased risk of both HPV infection and cervical cancer, accompanied by elevated IL-10 expression levels (106). Likewise, variants in *IL4* rs2243250 (−59° C > T), *IL-6* rs1800796 (−573 G > C), *IL-10* rs1800871 (−819 C > T), and *TGF-β1* rs1800469 (−509 C > T) have been associated with an increased CC susceptibility, with evidence suggesting that multiple risk alleles may exert additive effects (36).

Genetic associations have also been identified in other immune regulatory pathways. Variants in *IL-1B* (rs16944 and rs1143627) have been associated with cervical cancer susceptibility in Chinese populations (185, 186), whereas the TNF rs1800629 (−308 G > A) polymorphism has been linked to altered disease risk in Polish, Tunisian, and Nigerian populations (187–190). Similarly, the *IFN-γ* rs2430561 (+874 T > A) variant has been associated with CC susceptibility in Indian women and is thought to influence the ability to mount effective antiviral immune responses (191). In addition, polymorphisms in *IL-17A* (rs2275913) and *IL-17F* (rs763780) have been associated with increased CC risk in Chinese populations, further supporting a potential role for dysregulated Th17-mediated inflammation in HPV persistence and disease progression (192).

Evidence from meta-analyses and systematic reviews reinforces the contribution of cytokine-related genetic variants to cervical carcinogenesis, particularly polymorphisms in *IL-1B, IL-10, TNF, IFN,* and *TGF-β1*, although the magnitude of these associations varies across different ethnic populations (193, 194). Overall, these findings indicate that inherited variation in cytokine signaling pathways contributes to interindividual differences in antiviral immunity, viral persistence, immune evasion, and susceptibility to cervical cancer by shaping the inflammatory and immunoregulatory landscape of the host.

## 9 MicroRNA-mediated regulation of HPV persistence and cervical carcinogenesis

MicroRNAs (miRNAs) are small non-coding RNAs, approximately 22 nucleotides in length, that regulate gene expression through sequence-specific interactions with the 3′ untranslated regions (3′UTRs) of target mRNAs, resulting in translational repression or mRNA degradation (195). Although they constitute only a small fraction of the genome, miRNAs regulate a large number of protein-coding genes and participate in diverse biological processes, including cell proliferation, apoptosis, differentiation, metabolism, and immune regulation (196). In cervical cancer, persistent HPV infection disrupts miRNA expression, contributing not only to malignant transformation but also to immune evasion and remodeling of the tumor microenvironment.

Among the best-characterized oncogenic miRNAs, miR-21 is consistently overexpressed in cervical intraepithelial lesions and invasive cervical cancer. By repressing tumor suppressors such as PTEN and RECK, miR-21 promotes oncogenic signaling pathways that enhance cell proliferation, survival, migration, and invasion. In addition, miR-21 has been implicated in the regulation of inflammatory and immunosuppressive pathways, thereby linking tumor progression to immune dysregulation. Its expression is further enhanced by the AP-1 transcription factors c-Fos and c-Jun, which directly activate the miR-21 promoter in cervical cancer cells (197).

Conversely, several tumor-suppressive miRNAs are progressively downregulated during cervical carcinogenesis. Members of the miR-23/27/24 cluster, particularly miR-23b-3p, inhibit tumor growth and invasion by targeting the oncogene c-Met, whereas the miR-15/16 family restrains cell-cycle progression. Reduced expression of miR-16-1 is associated with increased CCNE1 expression and unfavorable clinical outcomes, highlighting the contribution of these miRNAs to malignant progression (198–201).

Epigenetic mechanisms further contribute to miRNA dysregulation during HPV-associated carcinogenesis. For example, hypermethylation of the miR-218 promoter in HPV16-positive cervical cancer reduces its expression, illustrating how persistent HPV infection may reshape the epigenetic landscape to alter miRNA-mediated gene regulation (202).

These findings demonstrate that dysregulated miRNA networks contribute to cervical carcinogenesis by coordinating oncogenic signaling, cell-cycle regulation, and immune modulation. Their dual roles in tumor biology and immune regulation also highlight their potential as biomarkers of disease progression and as targets for novel therapeutic strategies.

## 10 Mechanisms of immune evasion and therapeutic implications

Persistent HR-HPV infection is the essential etiological event in cervical carcinogenesis; however, malignant progression depends not only on viral persistence but also on the progressive establishment of an immunosuppressive tumor microenvironment. HPV orchestrates a coordinated immune-evasion program that disrupts innate immune sensing, antigen presentation, co-stimulatory signaling, and cytotoxic lymphocyte function, thereby creating a self-reinforcing niche that supports viral persistence and tumor progression (20, 203).

Immune evasion begins early through suppression of innate antiviral defenses. HPV early proteins E1, E2, E4, and E5 inhibit the cGAS–STING–TBK1–IRF3 axis and attenuate type I interferon responses (204). At the same time, E5 downregulates surface MHC-I expression (56, 205), whereas E6 and E7 interfere with Toll-like receptor signaling, antigen-processing machinery, and interferon pathways, collectively impairing dendritic cell activation and CD8⁺ T-cell priming (20, 203, 204, 206–208).

Persistent infection subsequently drives the establishment of an IL-10 and TGF-β1-dominated microenvironment. By transcriptionally enhancing the expression of these cytokines, HPV oncoproteins generate autocrine and paracrine feedback loops that inhibit dendritic cell maturation, reduce CD40, CD80, and CD86 expression, impair adaptive immunity, and promote long-term immune tolerance (34, 99, 103, 209). The restoration of dendritic cell function following IL-10 or TGF-β1 blockade further highlights these cytokines as promising therapeutic targets (210).

Defective adaptive immunity is further reinforced through disruption of the CD40/CD40L co-stimulatory pathway. HPV suppresses CD40 signaling indirectly through IL-10 and TGF-β1 and directly through E6-mediated degradation of the transcription factor AKNA, thereby impairing dendritic cell licensing and effective T cell activation (162, 211–214). Experimental evidence demonstrating enhanced antitumor immunity following CD40 agonism, together with the association between preserved CD40/CD40L expression and favorable clinical outcomes, supports this pathway as an attractive therapeutic target (215–217).

Sustained antigen exposure within this immunosuppressive environment progressively compromises both adaptive and innate immune responses. T cells transition from functional impairment to terminal exhaustion, characterized by reduced proliferation, diminished cytokine production, and co-expression of multiple inhibitory receptors, including PD-1, TIGIT, TIM-3, LAG-3, and NKG2A (150, 213, 218). Likewise, NK cells display reduced cytotoxicity as a result of the combined effects of diminished activating receptor expression, enhanced inhibitory signaling, soluble MICA, MICB, and B7-H6, chronic exposure to IL-10 and TGF-β1, hypoxia, and Fas-mediated apoptosis, although their residual functional plasticity may still be therapeutically exploitable (143, 150, 219–221). In parallel, expansion of regulatory T cells, activation of the Fas/FasL pathway, and accumulation of extracellular adenosine further reinforce local immune suppression (117, 181, 222–224).

Collectively, these findings support a hierarchical model of HPV-mediated immune escape in which early suppression of innate immune recognition is followed by progressive impairment of antigen presentation, co-stimulatory signaling, and cytotoxic lymphocyte function. Rather than acting independently, these complementary mechanisms cooperate to establish a self-perpetuating immunosuppressive microenvironment that sustains viral persistence and drives malignant progression (20, 203, 225).

This integrated model has important therapeutic implications. Although immune checkpoint inhibitors have improved outcomes in recurrent and metastatic cervical cancer, HPV-induced immune dysfunction extends well beyond checkpoint signaling (218, 226). Consequently, durable clinical responses will likely require rational combination strategies capable of restoring antigen presentation, enhancing CD40 signaling, targeting IL-10 and TGF-β1 pathways, reversing T- and NK-cell exhaustion, modulating regulatory T-cell activity, and strengthening HPV-specific immunity. Experimental evidence from diverse immunomodulatory strategies, including cytokine blockade, agonistic CD40 antibodies, soluble CD40L, selective inhibition of diacylglycerol kinase α, and adoptive transfer of HPV16 E6/E7-specific cytotoxic T lymphocytes, suggests that coordinated reprogramming of the immune response may achieve greater therapeutic efficacy than checkpoint inhibition alone (210, 216, 222, 227–229).

Overall, cervical cancer should be viewed as the consequence of progressive immune surveillance failure orchestrated by persistent HPV infection rather than as the result of isolated defects in antiviral immunity.

HPV vaccination: prophylactic vaccines, current limitations, and therapeutic perspectives.

### 11.1 Prophylactic HPV vaccines

HPV vaccination is the cornerstone of primary cervical cancer prevention and a central component of the World Health Organization (WHO) strategy to eliminate cervical cancer as a public health problem through the 90-70-90 targets, which aim to achieve 90% HPV vaccination coverage, 70% screening coverage, and 90% access to appropriate treatment by 2030 (230). Currently available bivalent, quadrivalent, and nonavalent vaccines are based on recombinant L1 virus-like particles (VLPs) that induce neutralizing antibody responses substantially stronger than those generated by natural HPV infection (231, 232). The bivalent vaccine targets the oncogenic HPV16 and HPV18 types, whereas the quadrivalent vaccine additionally targets HPV6 and HPV11, which are associated with most anogenital warts. The nonavalent vaccine further extends protection to the oncogenic HPV31, HPV33, HPV45, HPV52, and HPV58 types, thereby providing broader protection against HPV-associated cervical disease (224, 225). The composition of these vaccine types is well established in the literature and current vaccine guidance (233).

### 11.2 Clinical efficacy and immune responses to prophylactic vaccination

Large clinical trials have consistently demonstrated excellent efficacy against persistent HPV infection and cervical precancer. The quadrivalent vaccine provides approximately 98% protection against HPV16/18-associated cervical intraepithelial neoplasia grade 2 or worse (CIN2^+^) while also preventing HPV-related anogenital disease, whereas the bivalent vaccine confers sustained protection against cervical intraepithelial neoplasia grade 3 or worse (CIN3^+^) and adenocarcinoma *in situ* (234–237). Similar benefits have been reported in Mexican women, in whom the quadrivalent vaccine provided complete protection against HPV6-, HPV11-, HPV16-, and HPV18-associated disease and demonstrated an excellent safety profile (238).

Natural HPV infection elicits measurable mucosal and systemic antibody responses, although their magnitude and prevalence are variable, whereas prophylactic HPV vaccination induces substantially stronger cellular responses and type-specific humoral immunity (238–242). Studies in Mexican women have demonstrated vaccination-associated changes in several immune-regulatory receptors. Colmenares et al. reported increased ILT2 expression on NK cells, CD3⁺CD56⁺ lymphocytes, and monocytes following immunization with the quadrivalent HPV6/11/16/18 vaccine (243). *In vitro* stimulation with the vaccine also increased the proportion of CD3⁻CD56⁺ILT2⁺ NK cells. Although ILT2-mediated inhibition of cell proliferation was enhanced following vaccination, engagement of this receptor did not affect HPV-induced IFN-γ production. Vaccination was also associated with increased expression of the activating receptors NKG2D, NKp30, and NKp46 on NK cells and CD3⁺CD56⁺ lymphocytes (243).

Other studies in Mexican population have demonstrated the induction of HPV-specific CD8⁺IFN-γ⁺ T-cell responses (244), as well as antibody signatures capable of distinguishing vaccinated women from women with cervical lesions (241). In addition, cross-protection against phylogenetically related oncogenic HPV types supported the development of the nonavalent vaccine, which broadens protection against additional high-risk genotypes and is particularly valuable in regions with diverse HPV genotype distributions, including Latin America (245–248).

### 11.3 Population-level impact, vaccination schedules, and implementation challenges

The population-level impact of HPV vaccination has been substantial (232). A systematic review and meta-analysis of vaccination programs reported that, after 5–8 years of vaccination, HPV16/18 prevalence decreased by 83% among girls aged 13–19 years and by 66% among women aged 20–24 years. These reductions provide evidence of both direct vaccine effectiveness and herd effects at the population level (249). More recently, vaccination programs have also produced measurable reductions in invasive cervical cancer incidence (250).

Furthermore, evidence demonstrating that one- and two-dose schedules elicit durable immune responses comparable to those achieved with three doses has led the WHO to recommend simplified vaccination schedules for girls aged 9–14 years, improving both the feasibility and cost-effectiveness of immunization programs (251–255).

Beyond their established role in primary prevention, licensed HPV vaccines have also been investigated as an adjuvant strategy following excisional treatment of high-grade cervical lesions. Although these vaccines are not therapeutic and do not directly treat established CIN2+, vaccination administered around the time of conization may reduce the risk of subsequent or recurrent CIN2+. A recent systematic review and meta-analysis reported a lower risk of CIN2+ recurrence following perioperative HPV vaccination (256). However, a subsequent randomized-trial-focused reanalysis emphasized that the magnitude of this benefit may be influenced by biases inherent to observational studies and highlighted the need for adequately powered randomized trials to more precisely establish the effect of post-treatment vaccination (257).

Despite these remarkable advances, achieving cervical cancer elimination remains challenging in many low- and middle-income regions. Although most countries in Latin America and the Caribbean (LAC) have incorporated HPV vaccination into their national immunization programs, vaccination coverage remains heterogeneous and declined substantially in Mexico following the COVID-19 pandemic (254, 258, 259). Persistent barriers, including vaccine hesitancy, misinformation, HPV-related stigma, logistical limitations, and inequitable access to healthcare, continue to limit vaccine uptake, particularly in rural, Indigenous, and socioeconomically disadvantaged communities, which also bear the highest burden of cervical cancer (230, 254, 260). These disparities are reflected in regional disease patterns, with cervical cancer incidence in LAC remaining approximately twice that observed in North America (261). Likewise, women living in rural, Indigenous, and marginalized regions of Mexico continue to experience disproportionately high cervical cancer mortality reflecting unequal access to vaccination, screening, early diagnosis, and treatment (225, 262–267).

Although gender-neutral vaccination further strengthens herd immunity and reduces the burden of HPV-associated cancers in males, maximizing vaccination coverage among girls remains the most cost-effective strategy in resource-limited settings (247, 249, 268, 269).

HPV vaccination represents one of the most successful cancer prevention interventions ever developed. However, achieving the WHO elimination goals will require not only high vaccine coverage but also equitable implementation of HPV-based screening, early diagnosis, and timely treatment through comprehensive cervical cancer control programs (230).

### 11.4 Limitations of prophylactic vaccination and rationale for therapeutic vaccines

Despite their effectiveness in preventing HPV infection and associated disease, currently available prophylactic vaccines have an important limitation: they are designed to prevent new infections and do not eliminate established HPV infections or provide therapeutic benefit for pre-existing precancerous or malignant lesions (270–273). This limitation is particularly relevant for individuals who have already acquired persistent HR-HPV infection. In addition, insufficient vaccine coverage in many regions limits the population-level benefits of prophylactic vaccination. Together, these biological and implementation-related limitations have stimulated the development of therapeutic HPV vaccines designed to eliminate HPV-infected and transformed cells by inducing antigen-specific cellular immune responses (274–276).

The HPV oncoproteins E6 and E7 are particularly attractive targets for therapeutic vaccination because they are constitutively expressed in HPV-associated precancerous and malignant cells and play essential roles in maintaining the transformed phenotype (271, 272, 277). E6 promotes p53 degradation through its interaction with the E6-associated protein (E6-AP) ubiquitin ligase, whereas E7 disrupts pRb function, interfering with cell-cycle regulation and promoting uncontrolled proliferation (39, 41, 42). Because sustained expression of E6 and E7 contributes to the survival and proliferation of HPV-transformed cells, these viral oncoproteins provide targets for antigen-specific cellular immune responses.

### 11.5 Therapeutic vaccine platforms and clinical evidence

Several therapeutic HPV vaccine platforms are currently under clinical or preclinical investigation, including DNA- and mRNA-based vaccines, viral and bacterial vectors, and peptide- or protein-based approaches (274, 276, 278, 279). Among DNA-based vaccines, VGX-3100 (bizalimogene ralaplasmid) encodes modified HPV16 and HPV18 E6 and E7 antigens. In a randomized, double-blind, placebo-controlled Phase IIb trial involving women with HPV16/18-positive CIN2/3, VGX-3100 induced significantly greater histopathological regression than placebo and was also associated with HPV16/18 clearance, providing clinical evidence that therapeutic DNA vaccination can induce both lesion regression and virological clearance in a subset of patients (276).

Peptide-based therapeutic vaccination has also been evaluated clinically. Early studies using synthetic long peptides spanning HPV16 E6 and E7 demonstrated robust vaccine-induced HPV16-specific T-cell responses in patients with cervical cancer (274, 275). Kenter et al. demonstrated robust immunogenicity in a Phase I study of patients with advanced cervical cancer, while Welters et al. showed induction of tumor-specific CD4⁺ and CD8⁺ T-cell immunity following vaccination (274, 275). A subsequent randomized, placebo-controlled study in women with HPV16-positive high-grade cervical squamous intraepithelial lesions further demonstrated the induction of HPV16-specific T-cell responses following synthetic long-peptide vaccination (280).

Viral-vector approaches have shown encouraging activity in cervical precancer. In a Phase II trial, Vvax001, a replication-incompetent Semliki Forest virus-based vaccine encoding HPV16 E6 and E7, induced histopathological regression to CIN1 or no dysplasia in 50% (9/18) of patients with HPV16-positive CIN3, while HPV16 clearance was observed in 63% (10/16) of evaluable patients (279). Additional investigational approaches include multi-epitope protein vaccines targeting HPV16 E6/E7 antigens (281) and bacterial-vector strategies using recombinant *Lactococcus lactis* (282).

Preclinical studies have demonstrated that mRNA vaccines encoding HPV E6 and E7 antigens can induce robust antigen-specific cellular immune responses and antitumor activity in HPV-associated tumor models (283, 284). Lee et al. (2023) demonstrated that an mRNA vaccine encoding non-oncogenic HPV16 E6 and E7 antigens induced E6/E7-specific cellular immune responses and antitumor activity in preclinical models (283). Similarly, the lipid nanoparticle-formulated mRNA vaccine mHTV-02, targeting HPV16 and HPV18 E6/E7, induced antigen-specific cellular and memory T-cell responses, increased CD8⁺ T-cell infiltration into tumors, and promoted tumor regression in preclinical models (284). These findings support mRNA vaccination as an emerging therapeutic strategy for HPV-associated malignancies, although its efficacy in cervical precancer or cervical cancer remains to be established in clinical trials.

Together, these platforms illustrate the diversity of strategies currently being explored to induce cellular immunity against HPV-infected and transformed cells.

### 11.6 Combination and immune-boosting strategies

Variability in clinical responses to therapeutic HPV vaccines has encouraged the evaluation of strategies designed to enhance vaccine-induced antitumor immunity. One strategy involves combining therapeutic vaccination with immune checkpoint inhibitors (such as pembrolizumab or atezolizumab) to counteract immunosuppressive mechanisms within the tumor microenvironment.

For example, the therapeutic DNA vaccine VB10.16 has been evaluated in combination with the PD-L1 inhibitor atezolizumab in patients with persistent, recurrent, or metastatic HPV16-positive cervical cancer (285). This approach is particularly relevant because effective antitumor immunity depends not only on generating HPV-specific T cells but also on overcoming mechanisms of immune suppression and exhaustion within the tumor microenvironment. Other approaches are exploring vaccines that combine prophylactic and therapeutic antigens, including L2- and E7-based strategies, with the aim of simultaneously broadening protection against HPV infection and inducing cellular responses against HPV-transformed cells (286).

A persistent challenge across therapeutic vaccine studies is the absence of well-established immunological correlates that reliably predict clinical response. Although HPV-specific immune responses have been observed across therapeutic vaccine studies, validated immunological correlates that reliably predict histological regression or viral clearance have not yet been established (287). Future studies should therefore integrate the characterization of HPV-specific T-cell responses with virological, histological, and survival outcomes to identify biomarkers that can guide patient selection and treatment strategies.

### 11.7 Clinical implications and future perspectives

At present, no therapeutic HPV vaccine has been licensed for the treatment of established HPV infection, cervical precancer, or cervical cancer (271). Although clinical studies have demonstrated HPV clearance and histopathological regression in subsets of patients, supporting the potential of therapeutic vaccination as a less invasive approach, further validation in larger randomized trials is required (276, 278, 279). Future clinical implementation will depend on establishing long-term efficacy and safety, identifying predictive biomarkers and immunological correlates, and optimizing combination strategies with other immunotherapies, including immune checkpoint inhibitors (285).

Overall, prophylactic and therapeutic HPV vaccines represent complementary strategies. Prophylactic vaccination remains essential for preventing HPV infection and reducing cervical cancer incidence, whereas therapeutic vaccination may help address persistent HPV infection and established HPV-associated lesions (232, 250, 271). Integrating effective prevention, screening, treatment access, and emerging therapeutic immunotherapies will be important for further reducing the global burden of cervical cancer.

## 12 Limitations

This narrative review has several limitations. A substantial proportion of the mechanistic evidence on HPV-mediated immune evasion and immune dysregulation derives from *in vitro* and experimental models that may not fully reproduce the complexity of persistent HR-HPV infection and the cervical immune microenvironment *in vivo*. In addition, the clinical evidence is heterogeneous in terms of study populations, HPV genotypes, disease stages, sample types, and analytical approaches, which may limit direct comparisons and the generalizability of individual findings. Much of the available human evidence is also observational or cross-sectional, limiting causal inference between immune alterations, HR-HPV persistence, and disease progression.

Finally, as a narrative review, no formal risk-of-bias assessment or quantitative evidence synthesis was performed. Therefore, the findings should be interpreted as an integrative overview of current evidence rather than a systematic assessment of effect sizes or certainty of evidence. Further longitudinal studies in diverse populations are needed to validate the clinical relevance of the mechanisms described and their contribution to HPV persistence and cervical disease progression.

## 13 Future perspectives and concluding remarks

Considerable progress has been made in elucidating how persistent HR-HPV infection promotes immune evasion through the coordinated actions of viral oncoproteins, host genetic susceptibility, immunosuppressive cytokines, defective antigen presentation, impaired co-stimulatory signaling, metabolic reprogramming, and dynamic changes in immune cell populations. Nevertheless, important questions remain regarding the temporal hierarchy of these events and their relative contributions to HPV persistence, lesion progression, therapeutic response, and clinical outcome. Addressing these questions will require the development of integrated immune signatures that combine cytokine profiles, T- and NK-cell phenotypes, regulatory T-cell abundance, soluble immune mediators, and biomarkers of antigen presentation rather than relying on individual biomarkers alone (150, 218, 221).

The immune alterations described throughout form an interconnected network involving viral, host, and microenvironmental factors are summarized in Figure 4.

**Figure 4:**
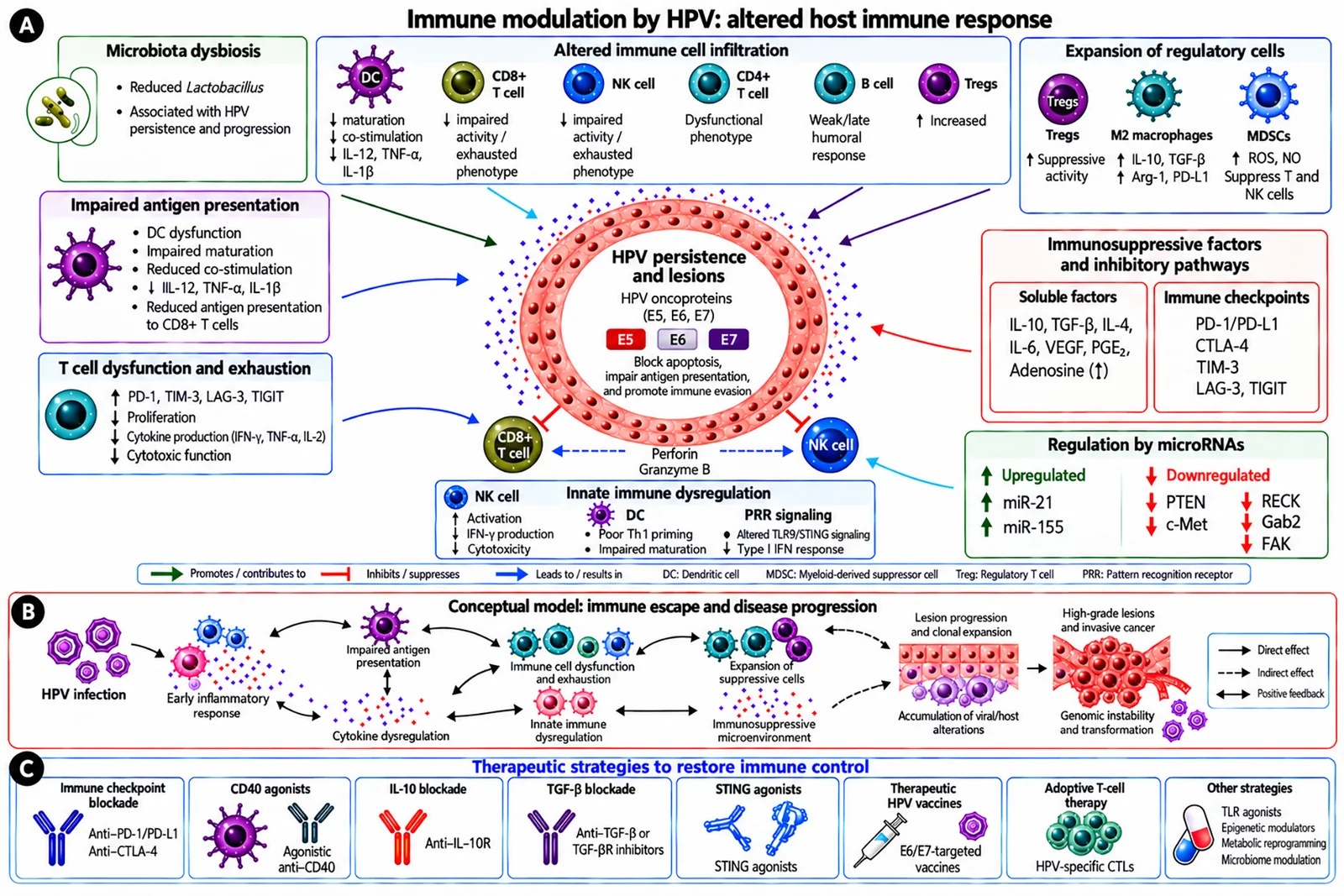
Altered immune response during persistent high-risk HPV infection. Persistent HPV infection reshapes the local immune microenvironment through microbiota dysbiosis, impaired antigen presentation, altered immune-cell infiltration, T-cell dysfunction, innate immune dysregulation, expansion of immunosuppressive cells, cytokine imbalance, and immune checkpoint activation, promoting viral persistence and lesion development **(A)** Panel B presents a conceptual model of the dynamic interactions among immune escape, chronic inflammation, viral persistence, lesion progression, and genomic instability that may contribute to cervical cancer, emphasizing that these processes are interconnected rather than strictly sequential. Panel C summarizes emerging therapeutic strategies aimed at restoring antitumor immunity and promoting viral clearance. This figure was created with the assistance of artificial intelligence (OpenAI ChatGPT/DALL·E) and subsequently reviewed and edited by the authors for scientific accuracy.

Rapid advances in single-cell and spatial transcriptomics, multiplex imaging, high-dimensional cytometry, and artificial intelligence (AI)-assisted multi-omics analyses are transforming our understanding of the cervical tumor microenvironment. These technologies enable the characterization of spatial and functional interactions among epithelial, stromal, and immune cell populations with unprecedented resolution. Integrating genomic, transcriptomic, epigenetic, immunological, imaging, and clinical datasets will facilitate the development of systems-level models capable of explaining interpatient heterogeneity, identifying clinically actionable immune vulnerabilities, and supporting precision medicine approaches for HPV-associated malignancies (288, 289). Emerging therapeutic platforms, including CRISPR-Cas9-based genome editing, also hold promise for the development of innovative strategies targeting HPV-associated cancers (290).

Collectively, the evidence reviewed here supports the concept that cervical cancer is not simply a consequence of persistent viral infection but rather the result of a progressive disruption of immune surveillance driven by persistent HR-HPV infection. Immune escape begins with suppression of innate antiviral sensing and antigen presentation and subsequently evolves into a complex immunosuppressive network characterized by defective co-stimulatory signaling, chronic cytokine dysregulation, expansion of regulatory immune populations, metabolic reprogramming, and functional exhaustion of T and NK cells. These interconnected processes ultimately establish a tumor microenvironment that favors viral persistence and malignant progression.

Translating this mechanistic knowledge into clinical practice represents the next major challenge. Future advances in cervical cancer control will depend on integrating immunological biomarkers into patient stratification, developing rational combination immunotherapies that simultaneously target multiple immune escape mechanisms, and identifying predictive markers of treatment response. At the population level, however, prophylactic HPV vaccination remains the most effective strategy for primary prevention, while HPV-based screening, early detection, and timely treatment of precancerous lesions continue to be indispensable for reducing disease burden. Ultimately, combining equitable vaccination programs, accessible screening and treatment services, precision immunotherapy, and biomarker-guided clinical decision-making offers the greatest opportunity to reduce the global burden of HPV-associated cervical cancer and accelerate progress toward its elimination.
